# Cobalt(II) Single‐Chain Magnet With Strong Anisotropy and Ferromagnetic IntraChain Coupling

**DOI:** 10.1002/chem.202503057

**Published:** 2025-11-28

**Authors:** Yongbing Shen, Mengxing Cui, Hiroyoshi Ohtsu, Olaf Stefanczyk, Masahiro Yamashita, Shin‐ichi Ohkoshi

**Affiliations:** ^1^ Department of Chemistry, School of Science The University of Tokyo Tokyo Japan; ^2^ School of Chemical Science and Engineering Tongji University Shanghai P. R. China; ^3^ Department of Chemistry School of Science Tokyo Institute of Technology Tokyo Japan; ^4^ Institute For Materials Research Tohoku University Sendai Japan; ^5^ DYNACOM (Dynamical Control of Materials)‐IRL2015, CNRS The University of Tokyo Tokyo Japan

**Keywords:** coordination polymer, correlation length, exchange coupling, magnetic anisotropy, single‐chain magnet

## Abstract

Co(II)‐based coordination polymer is reported as a new benchmark single‐chain magnet (SCM), exhibiting an energy barrier (*U*_eff_) of 560 K and a blocking temperature (*T*_B_) of 21.5 K‒both the highest values observed for SCM systems. The magnetic behavior originates from the synergistic balance between strong axial anisotropy (*D*_Co_ = –83 cm^−1^) and moderate ferromagnetic exchange (*J*_Co‐Co_ = +17.7 K), promoting long‐range spin correlation over thousands of magnetic ions. Detailed analysis of the correlation length and domain‐wall energy reveals a wide SCM regime and a 1D‐to‐3D magnetic crossover at low temperature. Unlike previously reported radical‐bridged SCMs, this work demonstrates a clean, intrinsic design strategy for realizing extreme single‐chain magnetism, offering new principles for developing robust molecular spin materials.

## Graphical abstract

A new high‐performance single‐chain magnet (*U*_eff_ = 560 K) exhibits strong magnetic anisotropy and ferromagnetic intrachain interactions using redox‐active bridging ligands.

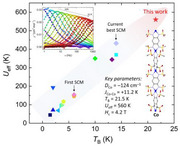

## 1 Introduction

Single‐chain magnets (SCMs) are molecular systems that exhibit slow magnetic relaxation along one‐dimensional (1D) chains, a phenomenon that emerges from the delicate interplay between strong intrachain magnetic exchange and significant magnetic anisotropy [1, 2, 3, 4, 5, 6, 7, 8, 9, 10]. Among various classes of SCMs, cobalt(II)‐based systems have attracted particular interest due to the inherently large anisotropy and flexible coordination geometries of Co(II) centers, making them promising candidates for achieving high blocking temperatures (*T*_B_) and large relaxation barriers (*U*_eff_) [11, 12, 13, 14, 15, 16, 17]. To date, many high‐performing Co(II)‐SCMs have relied on radical‐bridged architectures to enhance magnetic exchange and improve zero‐field magnetic hardness [18, 19, 20, 21, 22, 23]. While these systems, including early cobalt‐nitronyl nitroxide chains have demonstrated impressive coercivities (*H*_c_), their *T*_B_ and *U*_eff_ values remain relatively low (*T*_B_ < 10 K), and progress over the past decade has been limited [12]. Achieving SCMs with higher *T*_B_ and robust relaxation without relying on thermally unstable radical components remains a major challenge. Recent studies have suggested that it is possible to approach the intrinsic performance limits of Co(II)‐based SCMs through careful control of local coordination environments and inter‐ions coupling [11]. For example, Slageren and co‐workers reported that the strongly anisotropic Co(II) single‐molecule magnet (HNEt_3_)_2_Co(pdms)_2_ (*D*_Co_ = –115 cm^−1^, one of the largest negative *D* values reported so far),[24] which can be easily converted to a radical‐bridged dimer with significant inter‐SMM interactions, yielding excellent slow magnetic relaxation using the strongly electron‐donating ligand, 1,2,4,5‐tetrakis(methanesulfonamido)benzene (H_4_L) [25, 26, 27]. These preliminary works inspired us to construct a high‐performance SCM using such fantastic SMM units. In this work, we present a nonradical, undoped Co(II)‐based coordination polymer, [(HNEt_3_)_0_._5_Co(L)]_n_, which exhibits record‐setting SCM properties with *U*_eff_ = 560 K and *T*_B_ = 21.5 K, the highest values among all reported SCMs to date. This study diverges fundamentally from our earlier work on doped Co/Cu chains,[28] by shifting the focus to a pure Co(II) framework and thoroughly analyzing its intrinsic magnetic behavior, which was not fully studied in previous work. Our design leverages strong uniaxial anisotropy and moderate ferromagnetic exchange to balance domain‐wall energy and spin correlation growth, thereby stabilizing SCM behavior over a remarkably broad temperature window (Δ*T* ≈ 16 K). Furthermore, we provide a comprehensive physical description of the SCM‐to‐antiferromagnetic crossover, supported by temperature‐dependent correlation length (*ξ*_c_) analysis, hysteresis‐field scaling, and domain‐wall dynamics. This radical‐free platform not only pushes the performance limits of SCMs but also offers a robust design strategy for future spin‐based molecular devices, free from the complications of charge doping or electronic disorder.

**FIGURE 1:**
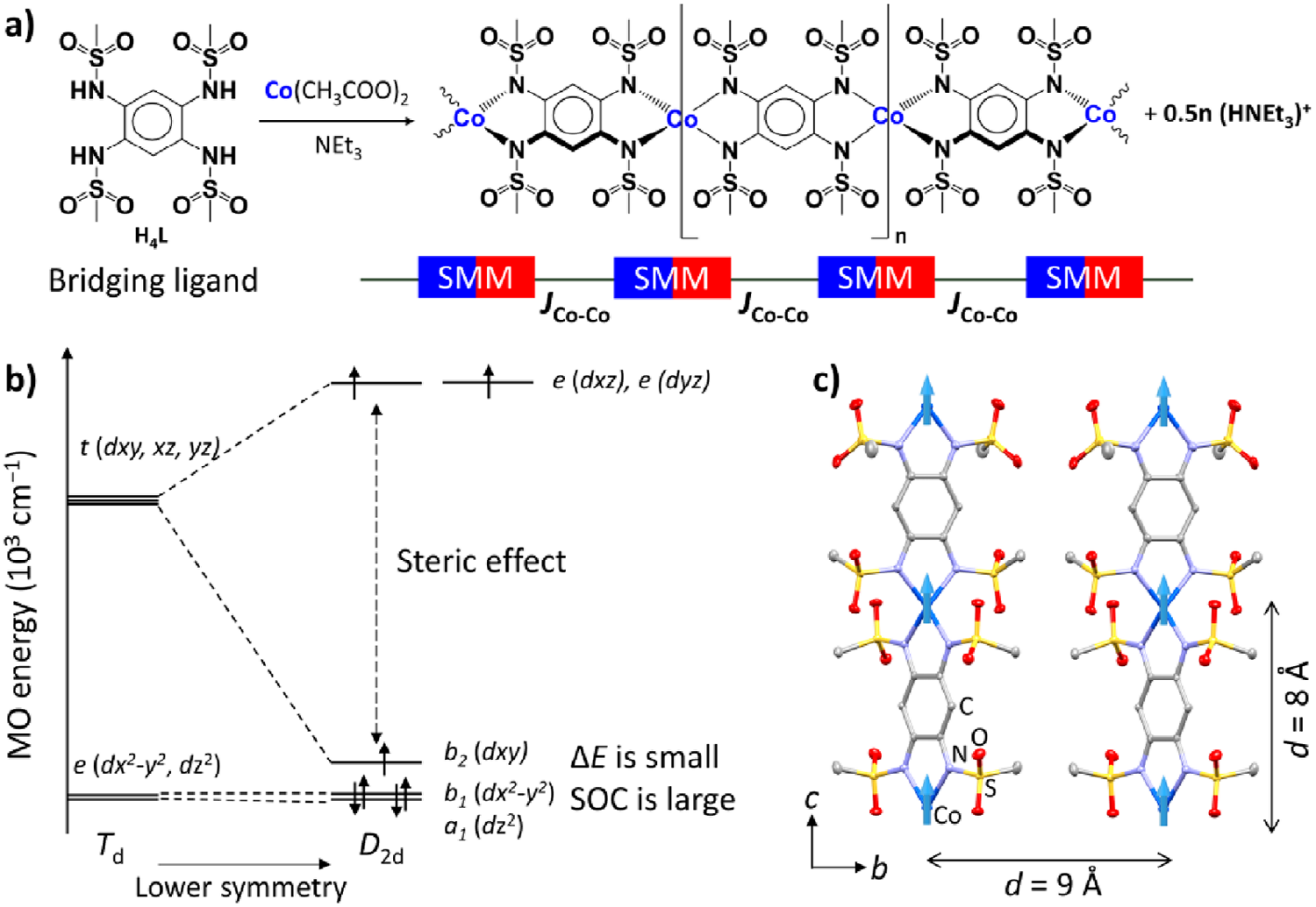
Molecular and electronic structure. a) Crystal packing viewed in the bc plane showing the 1D Co^2+^ chain; each Co^2+^ center is tetrahedrally coordinated by sulfonamidate N donors. b) Qualitative d‐orbital splitting diagram for Co^2+^ in an approximately *D*_2d_ ligand field within the **CoL** environment. c) Crystal structure in the *bc* plane highlighting the shortest interchain Co‐Co distance of 9 Å along the *b*‐axis. The hydrogen atoms were omitted for clarity. Color code: Co (blue), S (yellow), O (red), N (cyan), and C (gray).

We assembled linear Co^2^⁺ coordination chains by reacting the tetra‐sulfonamide bridging ligand H_4_L with Co(OAc)_2_ in the presence of NEt_3_, where deprotonated N donors anchor the metal nodes (Figure  1a). Each Co center displays strong axial anisotropy and SMM behavior, while adjacent Co sites are coupled through the ligand manifold, giving an effective intrachain superexchange *J*_Co–Co_​ that concatenates local SMM units into a one‐dimensional magnetic lattice. The Co centers adopt a tetrahedral coordination geometry due to the steric bulk of the bis(sulfonamido) substituents, which has been observed in many metal complexes [29, 30, 31]. The large *D* expected in this Co^2^⁺ complex originates from the relatively small energy gap (*ΔE*) between the nondegenerate a_1_ (*d*z^2^) and b_1_ (*d*xy) orbitals in the distorted *D*_2d_ crystal field (Figure 1b). This near‐degeneracy enhances spin–orbit coupling through low‐lying orbital excitations, particularly within the e(dx^2^−y^2^, dz^2^), leading to a significant contribution to the *D* value. The reduced symmetry of the coordination environment thus plays a crucial role in preserving unquenched orbital angular momentum and amplifying magnetic anisotropy [24, 32]. Crystal packing (Figure 1c) shows interchain separations of ∼9 Å along *b*‐axis and ∼8 Å along *c*‐axis, effectively suppressing dipolar and through‐space exchange between chains and enabling relaxation dynamics governed predominantly by intrachain interactions. Together, ligand‐induced geometric distortion and Co^2^⁺ SOC provide a coherent, multiscale design route (linking molecular orbitals, exchange pathways, and crystal architecture) to engineer anisotropy and *J*_Co–Co_ for high‐performance single‐chain magnetic materials.

## 2 Results and Discussion

**FIGURE 2:**
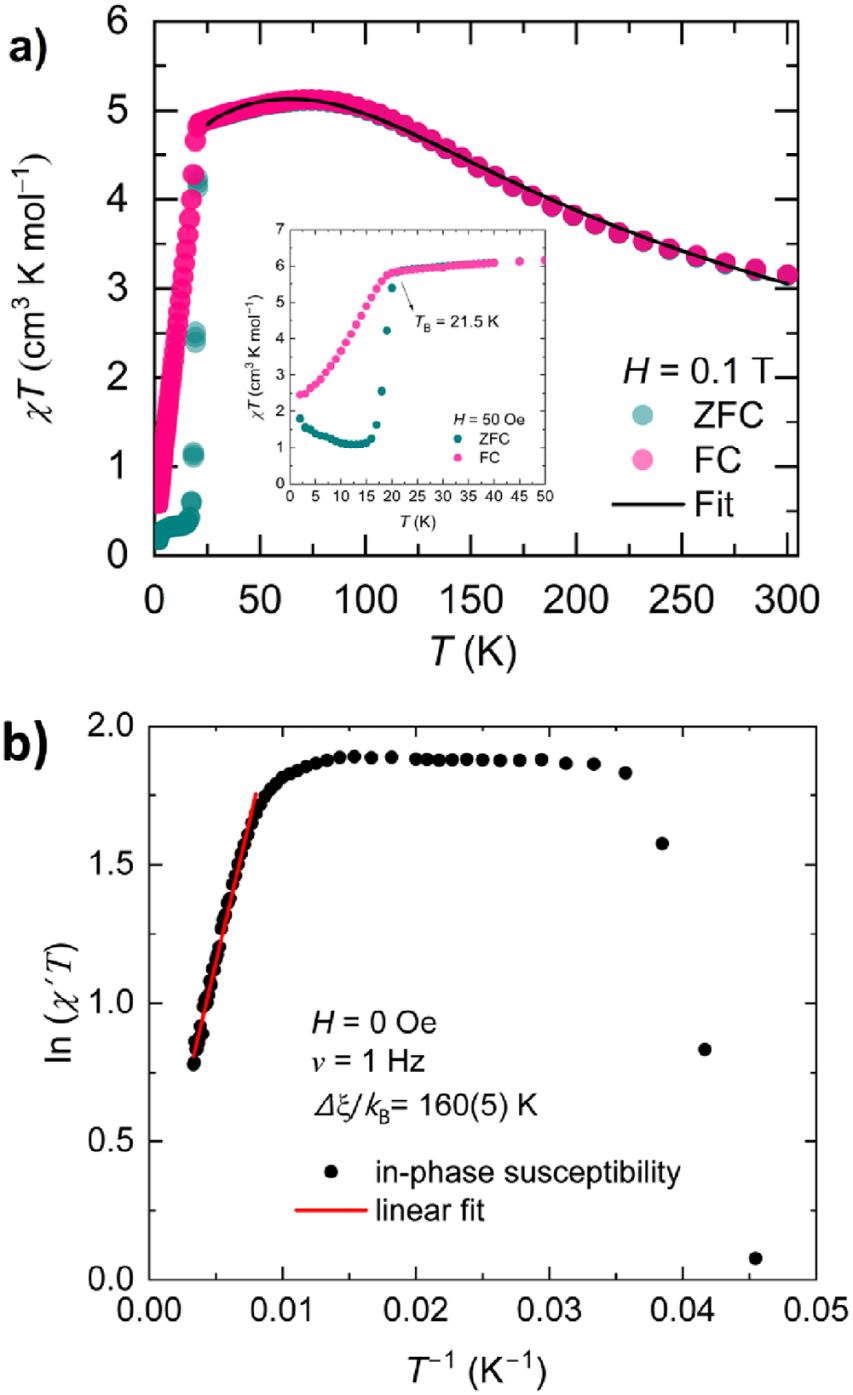
a) *χT* versus *T* measured at *H* = 0.1 T and *H* = 50 Oe. The comparable decrease of *χT* below ∼20 K at both fields indicates the downturn is intrinsic and not due to saturation at 0.1 T. Inset: ZFC/FC magnetization versus *T* at 50 Oe. b) The in‐phase susceptibility of ln (*χ′ T*) –(*T* ^‒1^) plot in 28–300 K at *H* = 0 Oe and 1 Hz, the red line is the fitting line to determine the domain‐wall energy.

The crystal structure was solved from the highly crystallized powders [28]. Growing single crystals is very difficult due to rapid polymerization in organic solvents. The chemical formula was determined as (HNEt_3_)_0.5_[Co(L)], where the L ligands showed partially oxidized states, which can be explained by strong electron donating ability in its deprotonated form (the first and second oxidization potential, *E_1/2_* = ‒1.1 V and ‐0.9 V *vs* Fc/Fc^+^). However, temperature dependence of ESR spectra of **CoL** indicated no magnetic contribution from organic spins (Figure S1). Therefore, **CoL** is treated as a pure high‐spin state Co(II) magnetic chain. The freshly prepared **CoL** powder was then used directly for magnetic measurements to preserve the integrity of the 1D chain structure, as mechanical grinding may disrupt spin correlation pathways. The temperature dependence of the direct current (*DC*) magnetic susceptibility (χT) is shown in Figure 2a. At 300 K, *χT* = 3.15 cm^3^ K mol^‒1^, consistent with a high‐spin Co^2+^ ion (*S* = 3/2) with *g* = 2.59. Upon cooling, *χT* steadily increases, reaching a broad maximum of 5.11 cm^3^ K mol^−^^1^ at 75 K, indicative of dominant intrachain ferromagnetic (FM) interactions. This behavior is reinforced by a large positive Curie–Weiss temperature (*θ*_CW_ = +75 K; Figure S2), reflecting the thermal development of intrachain spin coherence, in line with an Ising‐type ferromagnetic framework governed by anisotropic exchange. The origin of the relatively strong ferromagnetic interaction is due to several reasons. First, Co–O/N–(bridge)–O/N–Co orbitals are nearly orthogonal, antiferromagnetic (AF) superexchange is suppressed; second, spin polarization, the –O–SO_2_–N–SO_2_–O– bridge transmits same‐sign spin density; third, twist/low symmetry (*D*_2d_), steric twisting further weakens AF paths; finally, Co(II) has large spin orbital coupling and anisotropic exchange (*J*_zz_) favors parallel alignment. Below 21.5 K, a sharp decline in *χT* is observed, characteristic of domain‐wall freezing associated with slow magnetic relaxation. At 1.8 K, *χT* settles at 0.2 cm^3^ K mol^‒1^. Notably, a large bifurcation between zero‐field‐cooled (ZFC) and field‐cooled (FC) curves in a small, applied DC field (50 Oe) appears at *T*_B_ = 21.5 K (insert of Figure 2a), marking the onset of magnetic blocking. This value significantly exceeds the typical blocking temperatures (< 10 K) of most reported SCMs, including those employing radical bridges, and establishes **CoL** as a benchmark radical‐free system. Furthermore, ln(*χ′ T*)−*T* ^‒1^ plot (Figure 2b, Figure S3) revealed a linear dependence in 125−300 K, suggesting an Ising‐like 1D system [33, 34, 35]. Therefore, the energy for the creation of a domain wall, *∆*_ξ_/*k*_B_ = 160(5) K using

(1) $$\chi T\approx \;C_{eff}\times \exp\left(\Delta_{\xi }/k_{B}T\right)=2\xi_{c}/d$$

where *ξ*_c_ is the correlation length and *d* is the adjacent Co‐Co distance (*d* = 8 Å) along the chain. *ξ*_c_ is temperature‐dependent and expressed as 2*ξ*_c_ = 0.85 × exp(4*JS* ^2^/*T*) if *D*/*J* » 4/3. The *ξ*_c_ is about 1450 nm at *T*_B_ (4*JS* ^2^ = 160 K is used here), but it is still shorter compared to the physical chain length *ξ*_p_ (*ξ*_c_/*ξ*_p_ = 0.43). Thus, **CoL** can be treated as an infinite chain magnet [8]. To quantify the magnetic interactions above *T*_B_, the spin Hamiltonian (H) including the intrachain exchange coupling (*J*_Co‐Co_), magnetic anisotropy (*D*_Co_), and Zeeman effect is used to model magnetic dynamics:

(2) $$\begin{array}{ccc} H & = & \sum_{i=1}^{n}2J_{Co-Co}\left({\hat{S}}_{Co,i}\cdot {\hat{S}}_{Co,i+1}\right)+D_{Co,i}\sum_{i=1}^{n}{{\hat{S}}_{i}}^{2}\\ & & +\;\mu_{B}H\sum_{i=1}^{n}\left(g_{Co}\cdot {\hat{S}}_{i}\right) \end{array}$$

The best fit [36] between 25‒300 K yields *J*_Co−Co_ = +8(1) cm^‒1^, *D*_Co_ = ‒115 cm^−1^ (fixed), *g*_Co,⊥_= 2.20, *g*_Co,‖_= 2.98. The large negative *D*_Co_ value indicates the strong uniaxial magnetic anisotropy along the chains, which has been observed in the other reported SMMs [25]. As │*D*_Co_/*J*_Co‐Co_│= 14, the energy of the domain wall is expected as

(3) $$\Delta_{\zeta }/k_{B}=4J_{Co-Co}\cdot {S_{Co}}^{2}$$

**FIGURE 3:**
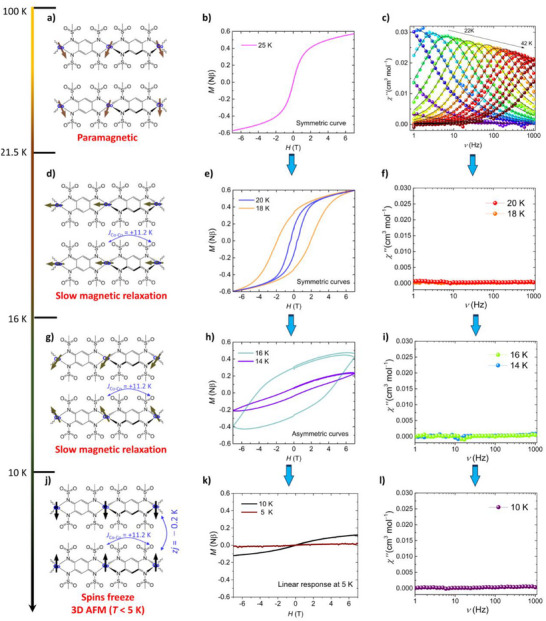
Spin Dynamics. a, d, g, j) Temperature dependence of spin patterns below 100 K. b, e, h, k) *M–H* curves in 5−25 K. c, f, i, l) Frequency dependence of out‐of‐phase magnetic susceptibility (*χ*") in 10−42 K, the black curves are the best fitting (Fitting equations in Figure S6).

Therefore, the *J*_Co‐Co_ = 160/(4×9/4) = +17.7 K, indicating the establishment of spin‐spin correlations within the chains below 160 K. The field dependence of magnetization (*M*–*H*) curves in 5‒25 K is shown in Figure S4. The temperature‐dependent evolution of magnetic hysteresis in SCMs is governed by the interplay between thermal fluctuations and the growth of intrachain spin correlations. At high temperatures (*T* > *T*_B_), thermal energy dominates, and the system exhibits a paramagnetic response with negligible spin *ξ*_C_ and zero *H*_C_, resulting in no magnetic hysteresis (Figures 3a and 3b). As the temperature decreases into the SCM regime (*T* < 21.5 K), spin–spin correlations along the chain increase substantially, leading to the

**FIGURE 4:**
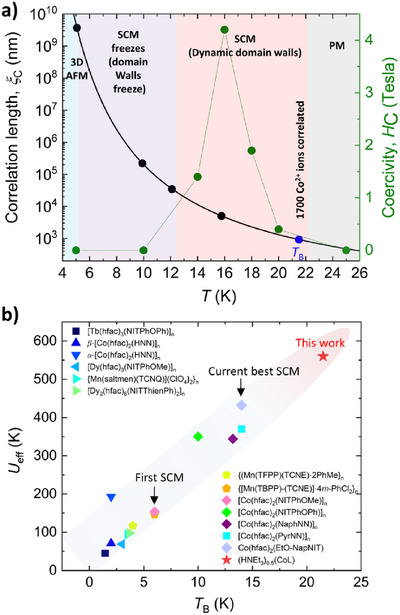
a) Magnetic phases of **CoL** in temperature‐dependent correlation length. The black line is the theoretical curve from equation (6), and the spots indicate the relationship between *ξ*_c_ and *ξ*_p_. b) The comparison of *U*_eff_ and *T*_B_ between **CoL** and reported radical‐bridged SCMs.

Development of extended magnetic domains. This gives rise to pronounced magnetic hysteresis with enhanced coercivity (Figures 3d, 3e, 3g, 3h). The maximum in *ξ*_C_, corresponding to several hundred Co^2^⁺ ions being magnetically correlated, reflects the formation of long spin segments acting as single magnetic entities (*H*_c_ up to 4.2 T and remaining magnetization (*B*_R_) of 0.3 Nβ at 16 K (Figure S5). However, upon further cooling below 16 K, domain walls become kinetically frozen due to insufficient thermal activation, resulting in a dramatic suppression of magnetization dynamics. This freezing behavior leads to a rapid decrease in coercivity and ultimately to the disappearance of the hysteresis loop (Figures 3j and 3k). Therefore, the observed nonmonotonic temperature dependence of the hysteresis width is a hallmark of dynamic domain wall motion in SCMs and reflects the temperature window in which slow magnetic relaxation and spin correlation are simultaneously optimized. Notably, the observed reduction in magnetization below 16 K can be primarily attributed to the freezing of domain walls in this SCM system. At temperatures above 16 K, magnetic *ξ*_C_ increases with decreasing temperature, allowing larger segments of the chain to respond coherently to the applied magnetic field. Around 16 K, *ξ*_C_ reaches the *ξ*_P_, and the chains behave as magnetically coherent units. However, further cooling below this point leads to a dramatic slowing of spin dynamics. The *U*_eff_ for domain wall motion becomes sufficiently large that spin reversal processes are hindered, resulting in frozen domain walls (Figure 4a). This leads to a suppression of magnetization, as spins can no longer realign effectively in response to the external magnetic field within the timescale of measurement. The *M*–*H* curve at 10 K exhibits a nearly paramagnetic‐like response with no noticeable hysteresis, suggesting that the system is in a crossover regime between the SCM state and a long‐range AFM ordered state. At this temperature, intrachain spin correlations remain strong, but interchain antiferromagnetic interactions begin to emerge, leading to suppressed magnetization without full ordering. The system still partially responds to external magnetic fields due to incomplete domain‐wall freezing and residual thermal activation, resulting in a nonlinear but reversible *M*–*H* profile. In addition to domain wall freezing, weak interchain AF interactions (*zj* = −0.2 K) may become nonnegligible at very low temperatures (*T* < 5 K) because the linear and hysteresis‐free *M*–*H* curve observed near 5 K suggests that the system undergoes a transition from a kinetically blocked SCM regime to a 3D AFM ordering (Figure 3k, T_N_ is also proved by observation of small peaks at 5 K from zero‐field *AC* data in Figure S3). In this regime, interchain AF interactions dominate, leading to a cancellation of the net magnetization and resulting in a linear response to the external magnetic field.

To gain deeper insight into the spin dynamics of **CoL**, alternating‐current (*AC*) susceptibility measurements were conducted. Above *T*_B_, **CoL** exhibits paramagnetic behavior with a strong frequency dependence in both the in‐phase (*χ*′) and out‐of‐phase (*χ*″) components (Figure 3c and *χ′* in Figure S6), indicating slow magnetic relaxation within the chains. When *T* < *T*_B_, the peaks of *χ′* and *χ″* shifted to much lower frequency (*ν* < 1 Hz), suggesting significant long relaxation times (Figures 3f, 3i, 3l, and Figure S7). The *χ*′*−χ*″ plots above *T*_B_ (21.5–42 K) confirmed single‐relaxation processes with *α* = 0–0.3 (Figure S8), and the temperature dependence of relaxation times (*τ*) follows Arrhenius‐type behavior (Figure S9), consistent with a single Orbach mechanism and suppression of Raman, direct, and tunneling processes. Fitting the *τ* with the Arrhenius law:

(4) $$\tau_{SCM}=\tau_{0}\exp\left(U_{eff}/T\right)$$

While *τ*_0_ = 2.4 ×10^−10^ s, comparable to values reported for mononuclear Co‐based SMMs,[24] the *U*_eff_ = 560 K notably surpasses all Co‐based SMMs/SCMs, establishing **CoL** as the highest‐performing SCM to date. To clarify the microscopic origin of the extremely large *U*_eff_, we applied the theoretical expression accounting for both domain‐wall ($\Delta_{\xi }$) and single‐ion anisotropy ($\Delta_{A}$) contributions:

(5) $$U_{\mathrm{eff}}=2\Delta_{\xi }+\Delta_{A}=8J_{\mathrm{Co}-\mathrm{Co}}\cdot S_{\mathrm{Co}}^{2}+\left|D_{\mathrm{Co}}\right|\left(S_{\mathrm{Co}}^{2}-1/4\right)$$

Using experimentally determined parameters (*S* = 3/2, *J* = +17.7 K, and *U*_eff_ = 560 K), the ZFS *D*_Co_ is determined as –83 cm^−1^. Approximately 43% of *U*_eff_ is derived from the local single‐ion anisotropy, while 57% originates from the energy cost of domain wall formation, emphasizing the synergistic contribution of microscopic anisotropy and collective spin correlation. The temperature dependence of *ξ*_c_, extracted from susceptibility measurements (Figure 4a), reveals a broad SCM regime between the PM and 3D AFM states. Notably, at *T*_B_ = 21.5 K, *ξ*_c_ already reaches 1450 nm (∼1700 Co^2^⁺ units), exceeding the full *ξ*_c_ of many radical‐bridged SCMs [12]. With further cooling, *ξ*_c_ increases exponentially, reaching a 504 nm (594 Co^2^⁺ ions) at 16 K. Below *T*_B_, the *H*_c_ is proportional to *ξ*_c_ and the largest *H*_c_ is generally observed when *ξ*_c_ equals the *ξ*_p_ (Figure 4a). Therefore, we estimated the *ξ*_p_ = 504 nm using the largest *H*_c_ (4.2 T) at 16 K. This behavior fits well when *T* is higher than *J*_Co‐Co_, with the expected 1D spin correlation expression:

(6) $$\xi \left(T\right)=\xi_{0}\text{exp}\left(\Delta_{\xi }/k_{B}T\right)$$

where *Δ*_ξ_/*k*_B_ = 4*JS* ^2^, highlighting the exponential growth of spin coherence as thermal fluctuations are suppressed. The breadth of the SCM regime (Δ*T* = 16 K) and the steep rise of *ξ*_c_ are rarely observed and provide strong evidence for a cooperative 1D‐to‐3D magnetic crossover. As illustrated in the *U*_eff_ *vs T*_B_ plot (Figure 4b), **CoL** (red star) represents the current state‐of‐the‐art, even compared to the radical‐bridged SCMs, achieving the highest combination of *U*_eff_ and *T*_B_. In contrast to other SCM systems where either low anisotropy or frustrated exchange limits performance, the rational design of **CoL** successfully integrates large uniaxial anisotropy, moderate ferromagnetic exchange, and extended spin correlations. These features collectively establish **CoL** as a new benchmark for SCM materials and demonstrate the power of coordination polymer engineering in advancing SCM.

## 3 Conclusion

In summary, **CoL** represents a new benchmark in Co(II)‐based SCMs, combining an exceptionally high *U*_eff_ and a record *T*_B_. The origin of this remarkable performance lies in the synergistic interplay between strong single‐ion anisotropy and moderate ferromagnetic exchange, which together promote extended intrachain spin correlation and a wide SCM regime. This work demonstrates that precise control over local magnetic parameters and polymer topology can yield unprecedented spin dynamics, offering valuable design principles for next‐generation molecular magnetic materials.

## 4 Experimental Sections

The synthesis and structural characterization of **CoL** are referred to the reported literature [28]. 0.05 mmol of crystallized cobalt acetate tetrahydrate, 0.05 mmol of crystallized H_4_L, and 20 mL of NEt_3_ were added to a 50 mL vial. The mixture was first stirred in open air for 10 min and then close the cap to stir for 3–5 days. The resulting dark blue‐violet solids were collected by filtration, washed with water and then large amounts of ethyl acetate, acetone, and finally dried at 60°C under vacuum. CHN elemental analysis: Calculated: C: 27.83%; H: 3.95%; N: 11.23%. Found: C: 27.17%; H, 4.62%; N, 10.83%; IR (KBr mode): ν(CH_3_) = 2932 cm^−1^; ν(S = O) = 1110 cm^−1^. CCDC No: 2132587.

Characterization: Magnetic susceptibility measurements were conducted on polycrystalline samples using a Quantum Design SQUID magnetometer MPMS‐7T. Diamagnetic corrections were estimated by Pascal's constants. AC measurements were performed in the frequency range of 1–1500 Hz by using SQUID.

## Conflict of Interest

The authors declare no conflict of interest.

## Supporting information




**Supporting File 1**: chem70486‐sup‐0001‐SuppMat.docx.

## Data Availability

The data that support the findings of this study are available in the supplementary material of this article.
