# Supplementary material for: Cobalt(II) Single‐Chain Magnet With Strong Anisotropy and Ferromagnetic IntraChain Coupling
Source: Chemistry. 2025 Nov 28;32(1):e03057. doi: 10.1002/chem.202503057 (PMC12759173; doi:10.1002/chem.202503057)
Supplement: Supplementary file 1 — Supporting File 1: chem70486‐sup‐0001‐SuppMat.docx. [file CHEM-32-e03057-s001.docx]

*Supporting information for:*

**Cobalt(II) Single-Chain Magnet With Strong Anisotropy and Ferromagnetic Intra-Chain Coupling**

Yongbing Shen,* Mengxing Cui, Hiroyoshi Ohtsu, Olaf Stefanczyk, Masahiro Yamashita,* and Shin-ichi Ohkoshi,*

Dr.Yongbing Shen, Prof. Shin-ichi Ohkoshi
Department of Chemistry, School of Science
The University of Tokyo
7-3-1 Hongo, Bunkyo-ku, Tokyo 113-0033, Japan
E-mail: [yongbingshen@g.ecc.u-tokyo.ac.jp](mailto:yongbingshen@g.ecc.u-tokyo.ac.jp); ohkoshi@chem.s.u-tokyo.ac.jp

Dr. Mengxing Cui; Prof. Masahiro Yamashita
School of Chemical Science and Engineering,
Tongji University
Siping Road 1239, Shanghai 200092, P. R. China

Dr. Hiroyoshi Ohtsu
Department of Chemistry, School of Science
Tokyo Institute of Technology
2-12-1 Ookayama, Meguro-Ku, Tokyo 152-8550, Japan

Prof. Masahiro Yamashita
Institute for Materials Research
Tohoku University
2-1-1 Katahira, Aoba-Ku, Sendai 980-8577, Japan
E-mail: [masahiro.yamashita.c5@tohoku.ac.jp](mailto:masahiro.yamashita.c5@tohoku.ac.jp)

Prof. Shin-ichi Ohkoshi
DYNACOM (Dynamical Control of Materials)-IRL2015, CNRS
The University of Tokyo
7-3-1 Hongo, Bunkyo-ku, Tokyo 113-0033, Japan
E-mail: ohkoshi@chem.s.u-tokyo.ac.jp


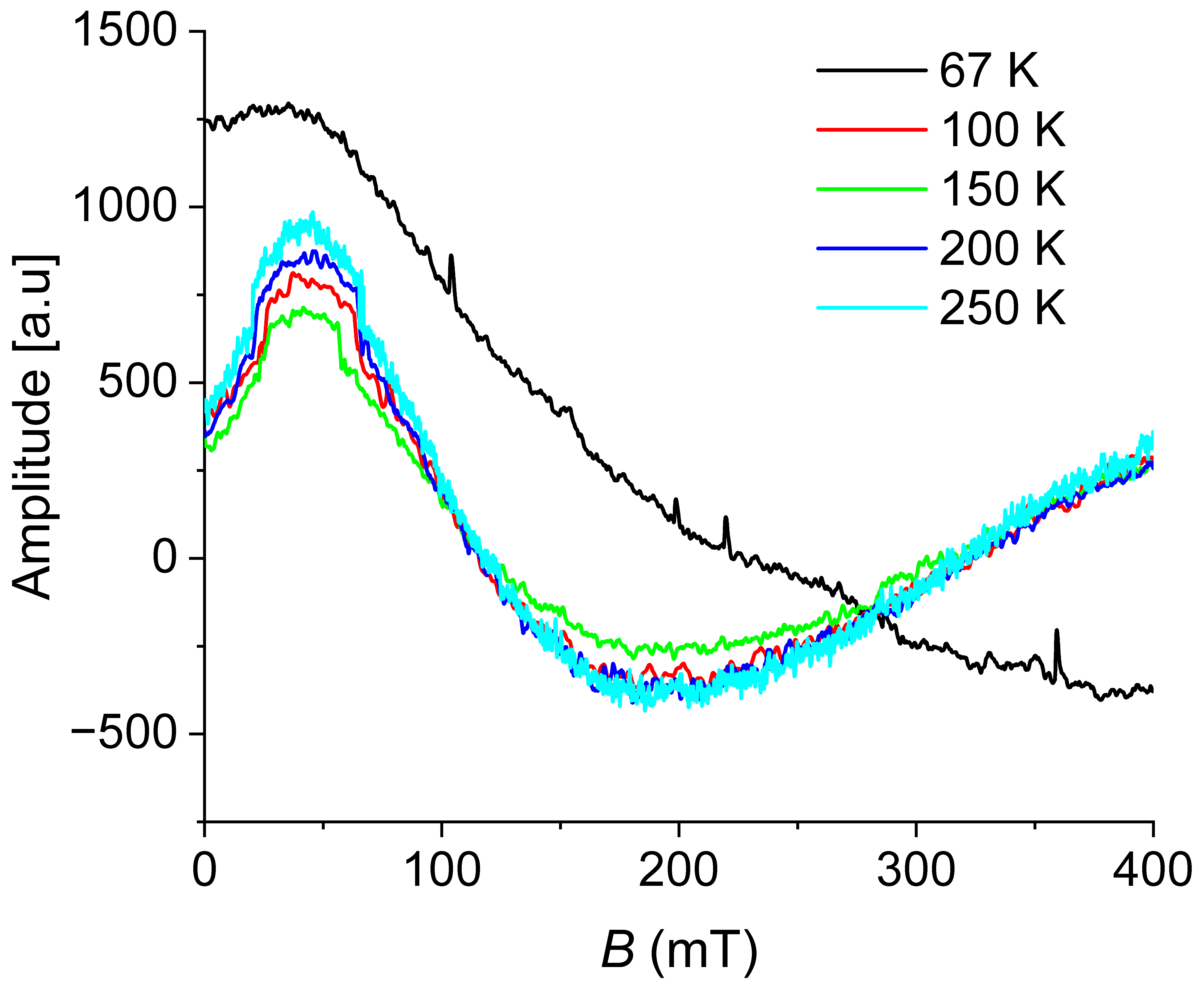


**Figure S1**. Temperature dependence of ESR spectra of **CoL**. No organic radicals’ signals are observed.


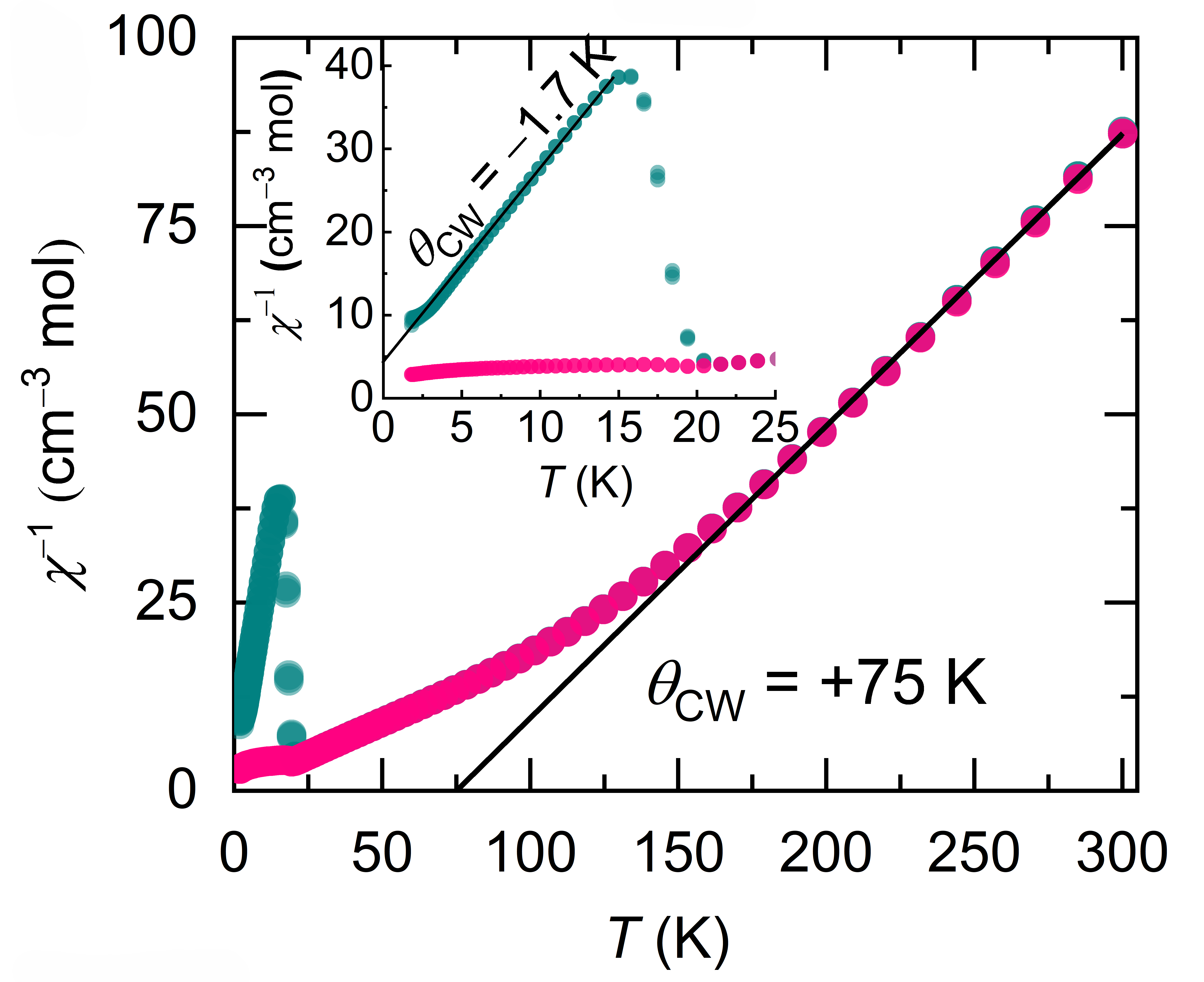


**Figure S2.** The *χ*^−1^−*T* plot (inset is the low temperature χ–1−T plot), the black line represents the best fit by Curie-Weiss law.


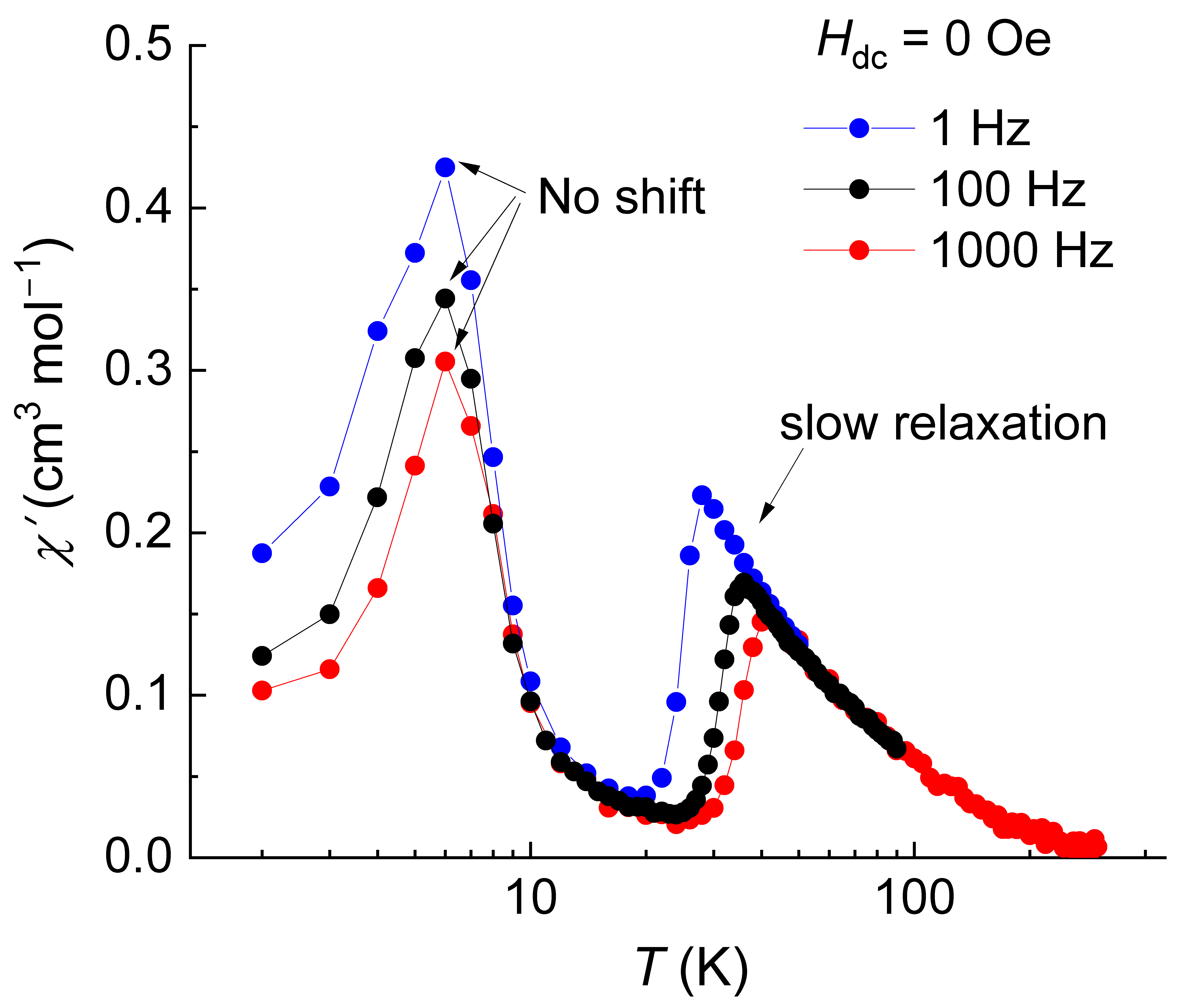


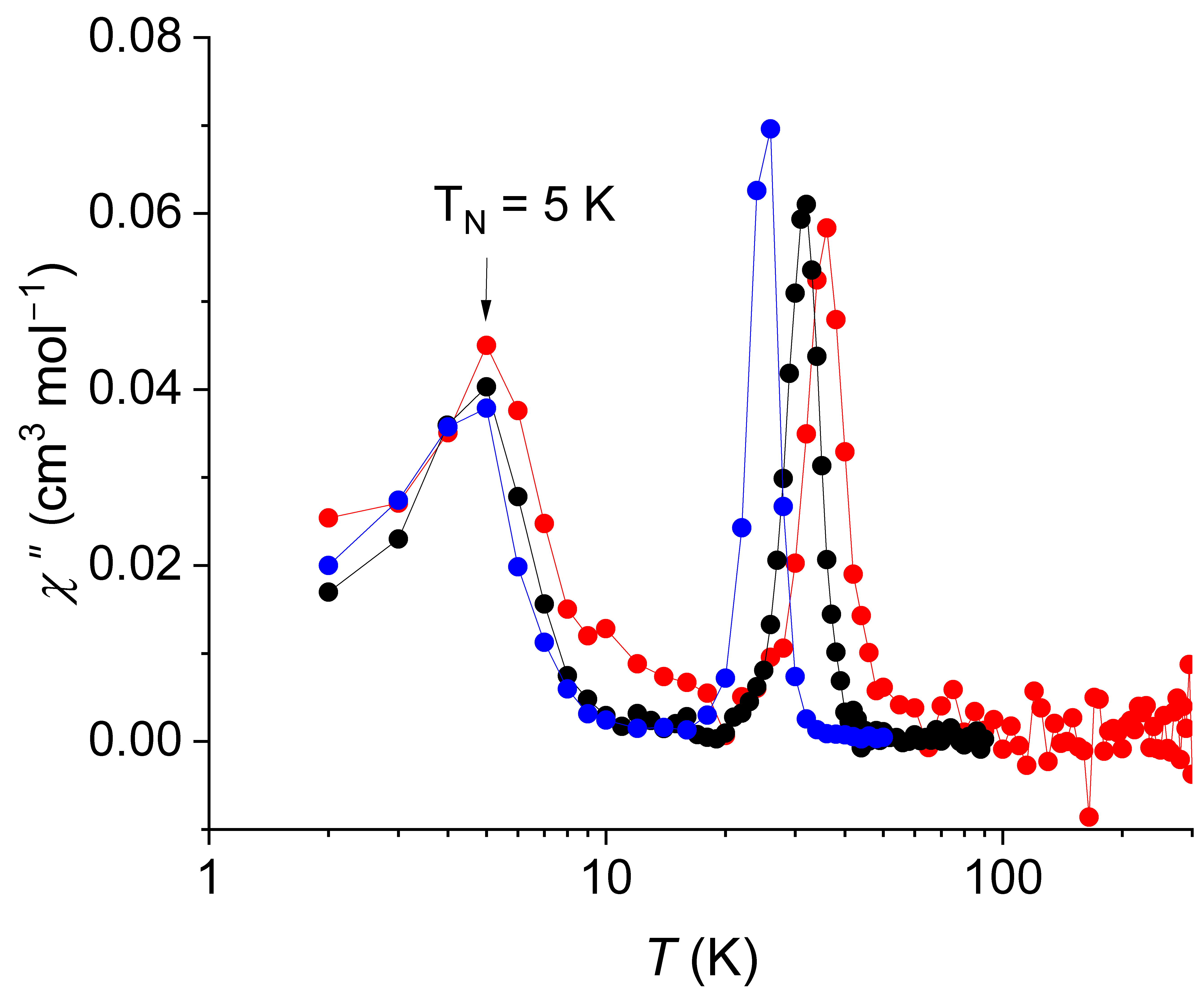


**Figure S3.** Temperature dependence of *AC* magnetic susceptibility in a zero-field and 100 Hz in 2-300 K.


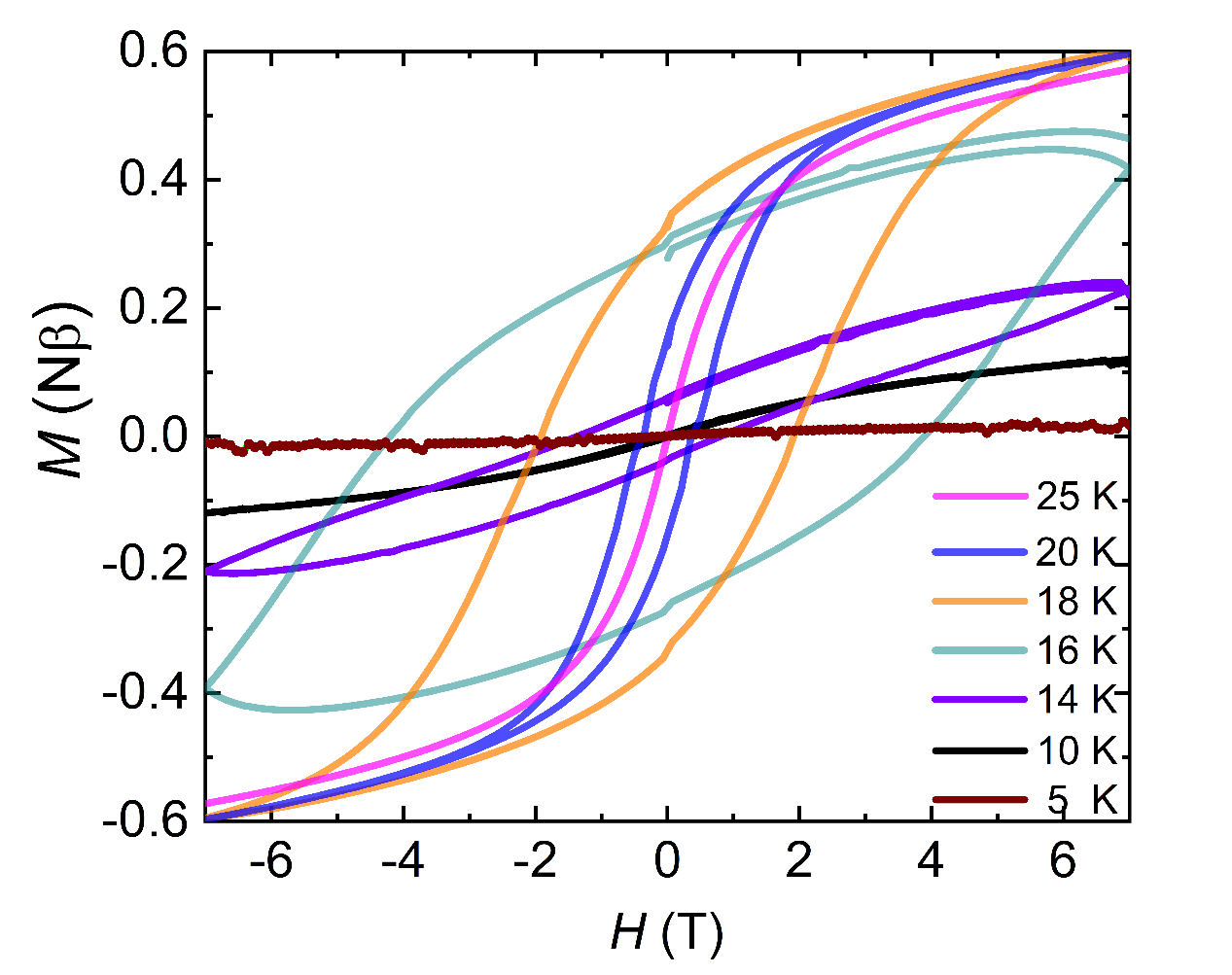


**Figure S4.** *M*-*H* curves in 2−25 K using SQUID with a static method.


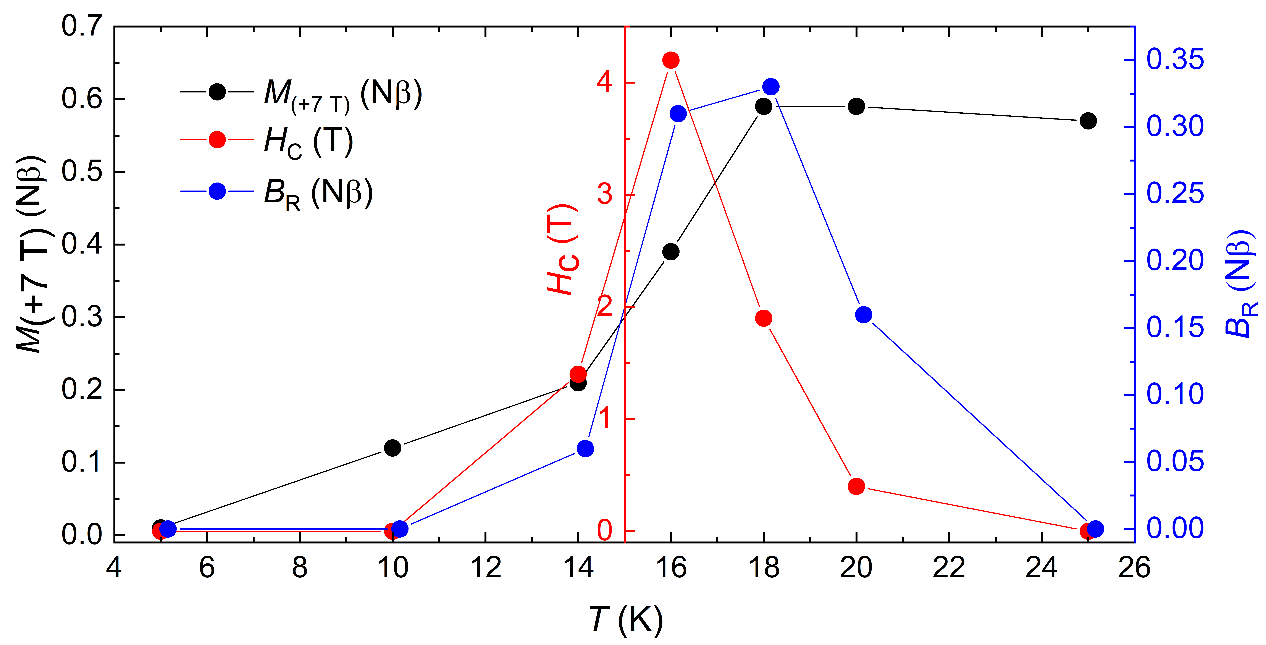


**Figure S5.** Temperature dependence of magnetization (*M*), Coverity (H_C_) and remaining magnetization (*B*_R_) in 4−26 K.


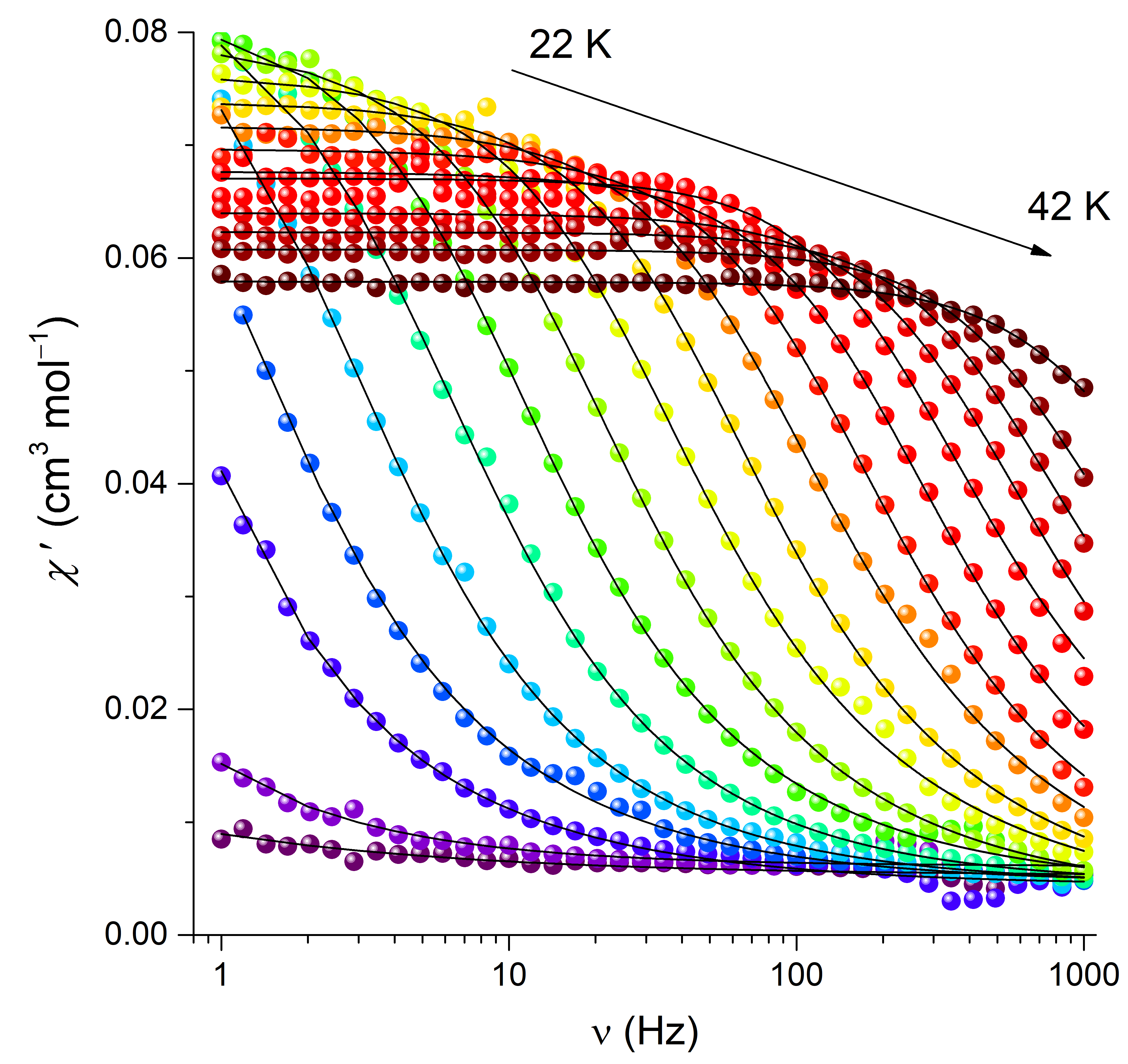


**Figure S6.** Frequency dependence of in-phase susceptibility *χ′* in 22−42 K, the black curves are the fitting. Fitting equations are shown as below:

**
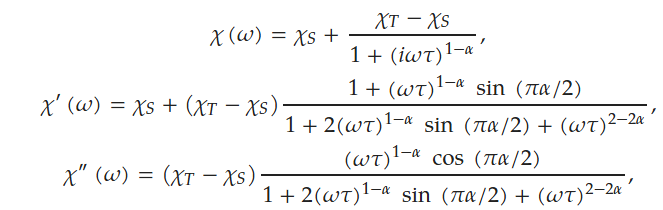
**


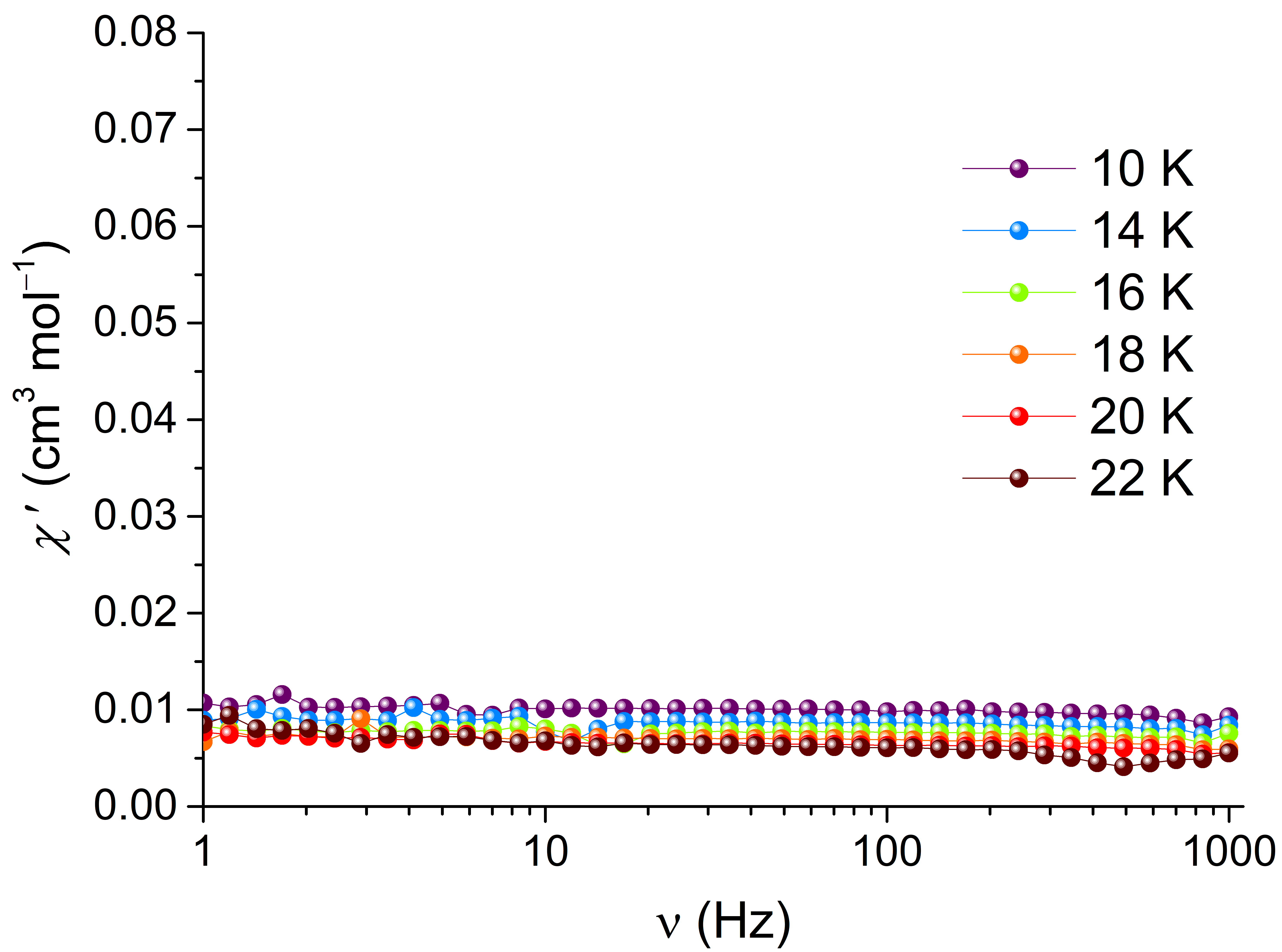


**Figure S7.** Frequency dependence of in-phase susceptibility *χ′* in 10−22 K.


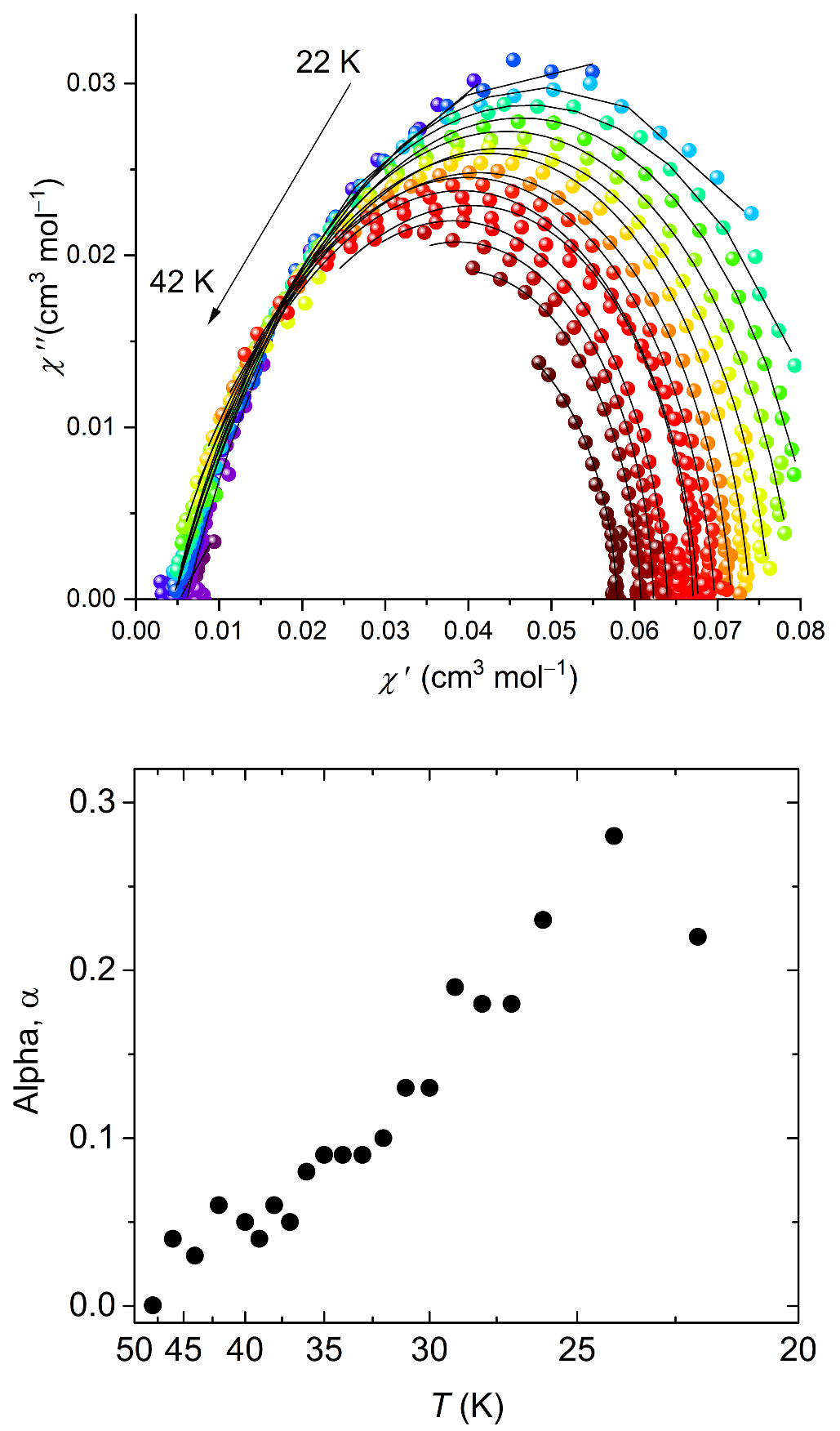


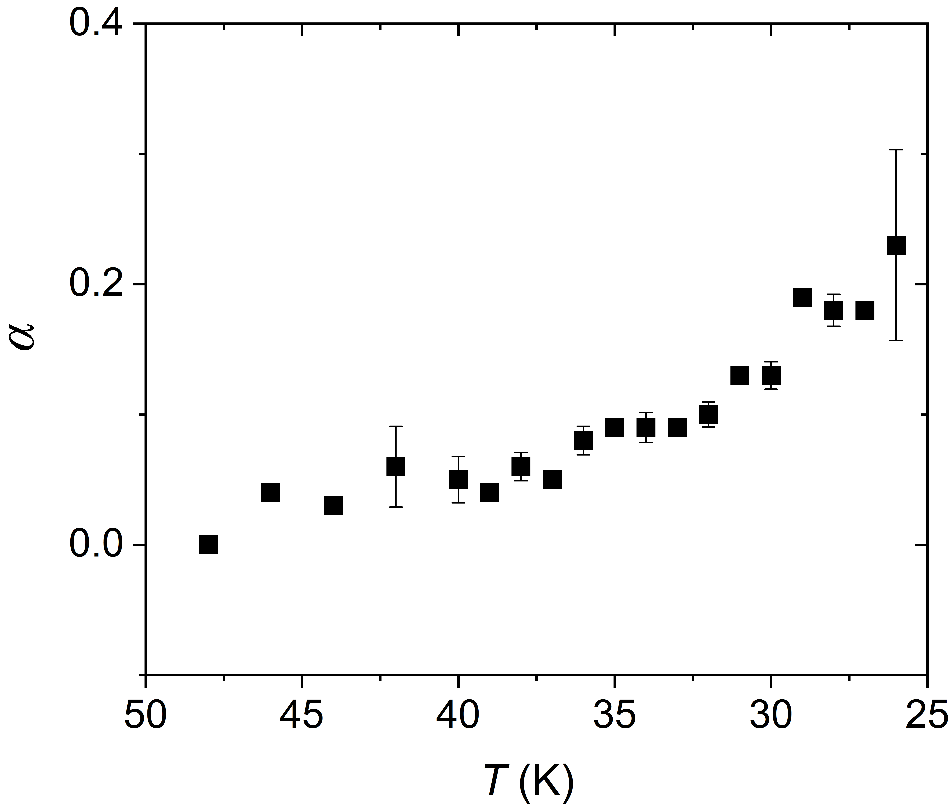


**Figure S8.** a) Cole-Cole plot in 22−42 K. b) the corresponding distribution parameter, alpha in 22−42 K, the value less than 0.3 indicates SCM behavior.


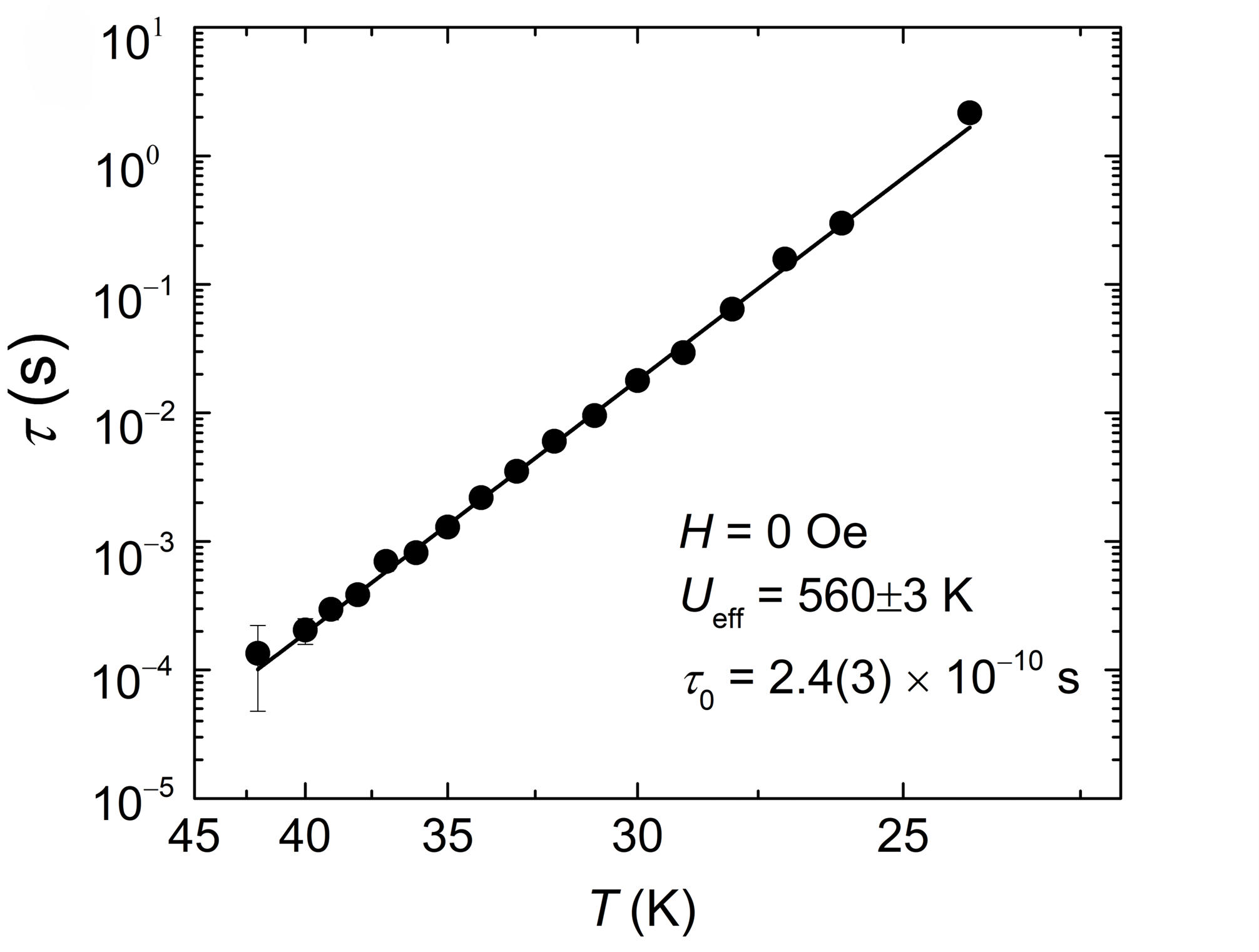


**Figure S9.** The temperature dependence of spin relaxation (*τ*) time in 22-42 K in 0 Oe. The fitting equation is used as equation (4) in the main text.


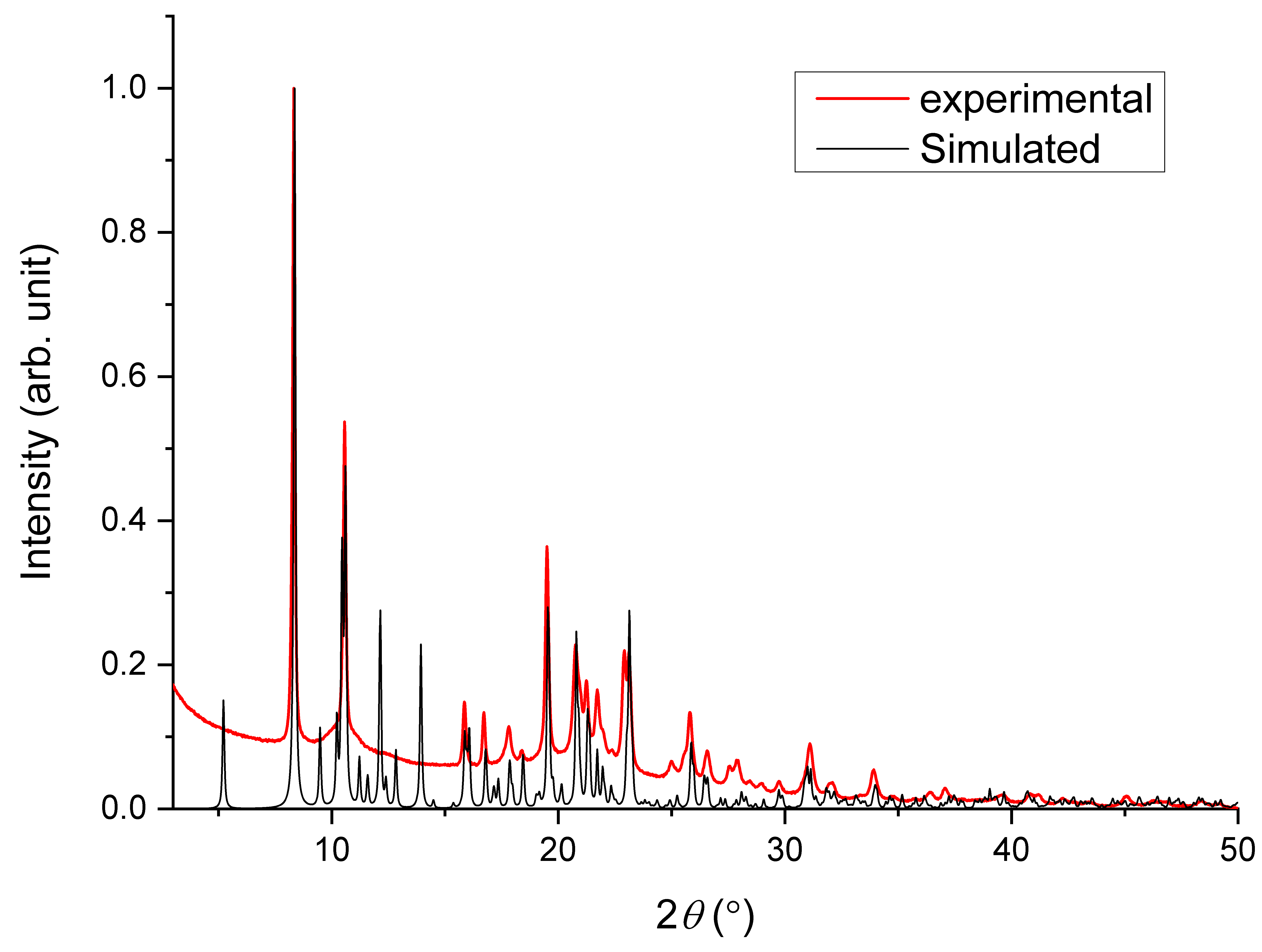


**Figure S10**. The experimental and simulated PXRD. Due to the difficulty of determining the crystal structure precisely from the PXRD pattern, the simulated and experimental PXRD patterns are not identical.
